# Small Bowel Adenocarcinomas Featuring Special AT-Rich Sequence-Binding Protein 2 (SATB2) Expression and a Colorectal Cancer-Like Immunophenotype: A Potential Diagnostic Pitfall

**DOI:** 10.3390/cancers12113441

**Published:** 2020-11-19

**Authors:** Giuseppe Neri, Giovanni Arpa, Camilla Guerini, Federica Grillo, Marco Vincenzo Lenti, Paolo Giuffrida, Daniela Furlan, Fausto Sessa, Erica Quaquarini, Alessandra Viglio, Cristina Ubezio, Alessandra Pasini, Stefano Ferrero, Gianluca Sampietro, Sandro Ardizzone, Giovanni Latella, Claudia Mescoli, Massimo Rugge, Fabiana Zingone, Valeria Barresi, Rachele Ciccocioppo, Paolo Pedrazzoli, Gino Roberto Corazza, Ombretta Luinetti, Enrico Solcia, Marco Paulli, Antonio Di Sabatino, Alessandro Vanoli

**Affiliations:** 1 Anatomic Pathology Unit, Department of Molecular Medicine, University of Pavia and Fondazione IRCCS Policlinico San Matteo, 27100 Pavia, Lombardy, Italy; giuseppe.neri01@universitadipavia.it (G.N.); giovanni.arpa90@gmail.com (G.A.); camilla.guerini01@universitadipavia.it (C.G.); a.viglio@smatteo.pv.it (A.V.); o.luinetti@smatteo.pv.it (O.L.); solciae@smatteo.pv.it (E.S.); m.paulli@smatteo.pv.it (M.P.); 2 Pathology Unit, Department of Surgical and Diagnostic Sciences, University of Genoa and Ospedale Policlinico San Martino University Hospital, 16132 Genoa, Liguria, Italy; federica.grillo@unige.it; 3 Department of Internal Medicine, University of Pavia and Fondazione IRCCS San Matteo Hospital, 27100 Pavia, Lombardy, Italy; marco.lenti@unipv.it (M.V.L.); paolo.giuffrida01@universitadipavia.it (P.G.); gastro@smatteo.pv.it (C.U.); a.pasini@smatteo.pv.it (A.P.); paolo.pedrazzoli@unipv.it (P.P.); gr.corazza@smatteo.pv.it (G.R.C.); a.disabatino@smatteo.pv.it (A.D.S.); 4 Pathology Unit, Department of Medicine and Surgery, University of Insubria, 21100 Varese, Lombardy, Italy; daniela.furlan@uninsubria.it (D.F.); fausto.sessa@uninsubria.it (F.S.); 5 Medical Oncology Unit, IRCCS ICS Maugeri and Experimental Medicine School, University of Pavia, 27100 Pavia, Lombardy, Italy; erica.quaquarini@icsmaugeri.it; 6 Division of Pathology, Fondazione IRCCS Ca’ Granda Ospedale Maggiore Policlinico, Department of Biomedical, Surgical and Dental Sciences, University of Milan, 20122 Milan, Lombardy, Italy; stefano.ferrero@unimi.it; 7 ASST Rhodense, Rho Hospital, 20017 Rho, Lombardy, Italy; gianluca.sampietro@unimi.it; 8 Gastroenterology Unit, Luigi Sacco University Hospital, 20157 Milan, Lombardy, Italy; sandro.ardizzone@fbf.milano.it; 9 Gastroenterology Unit, Department of Life and Environmental Sciences, University of L’Aquila, 67100 L’Aquila, Abruzzo, Italy; giolatel@tin.it; 10 Pathology Unit, Department of Medicine DIMED, University of Padua, 35121 Padova, Veneto, Italy; claudia.mescoli@libero.it (C.M.); massimo.rugge@unipd.it (M.R.); 11 Veneto Tumor Registry, 35121 Padova, Veneto, Italy; 12 Gastroenterology Section, Department of Surgery, Oncology and Gastroenterology, University of Padua, 35128 Padua, Veneto, Italy; fabiana.zingone@unipd.it; 13 Department of Diagnostics and Public Health, Section of Anatomical Pathology, University and Hospital Trust of Verona, 37126 Verona, Veneto, Italy; valeria.barresi@univr.it; 14 Gastroenterology Unit, Department of Medicine, AOUI Policlinico G.B. Rossi, University of Verona, 37134 Verona, Veneto, Italy; rachele.ciccocioppo@univr.it; 15 Oncology Unit, IRCCS San Matteo Hospital, 27100 Pavia, Lombardy, Italy

**Keywords:** celiac disease, Crohn’s disease, cytokeratin, mismatch repair, small intestine

## Abstract

**Simple Summary:**

Since small bowel adenocarcinoma may mimic a colorectal primary neoplasm histologically, it is pivotal to find biomarkers to discriminate these two biologically distinct neoplasms. The aim of our study was to evaluate the expression of special AT-rich sequence-binding protein 2 (SATB2), expressed in the vast majority of colorectal carcinomas, and other gastrointestinal phenotypic markers, such as cytokeratin 7, cytokeratin 20 and caudal type homeobox 2 (CDX2), in 100 small bowel adenocarcinomas. We identified 20 SATB2-positive small bowel adenocarcinomas, including nine sporadic cancers, seven celiac disease-associated cancers and four Crohn’s disease-associated small bowel adenocarcinomas. Six small bowel adenocarcinomas, including two cases associated with celiac disease and four sporadic, displayed a full colorectal carcinoma-like immunoprofile. Unlike SATB2, cytokeratin patterns stratified small bowel adenocarcinoma patient prognosis. The small bowel should be considered as one of the possible sites of origin in cancers of unknown primary, even when the neoplasm shows a colorectal carcinoma-like immunoprofile.

**Abstract:**

Special AT-rich sequence-binding protein 2 (SATB2) is a transcription factor expressed by colonic cryptic epithelium and epithelial neoplasms of the lower gastrointestinal (GI) tract, as well as by small bowel adenocarcinomas (SBAs), though at a lower rate. Nevertheless, up to now, only small SBA series, often including a very limited number of Crohn’s disease-associated SBAs (CrD-SBAs) and celiac disease-associated SBAs (CD-SBA), have been investigated for SATB2 expression. We evaluated the expression of SATB2 and other GI phenotypic markers (cytokeratin (CK) 7 and CK20, caudal type homeobox 2 (CDX2) and alpha-methylacyl-CoA racemase (AMACR)), as well as mismatch repair (MMR) proteins, in 100 SBAs, encompassing 34 CrD-SBAs, 28 CD-SBAs and 38 sporadic cases (Spo-SBAs). Any mutual association and correlation with other clinico-pathologic features, including patient prognosis, were searched. Twenty (20%) SATB2-positive SBAs (4 CrD-SBAs, 7 CD-SBAs and 9 Spo-SBAs) were identified. The prevalence of SATB2 positivity was lower in CrD-SBA (12%) in comparison with both CD-SBAs (25%) and Spo-SBAs (24%). Interestingly, six SBAs (two CD-SBAs and four Spo-SBAs) displayed a full colorectal carcinoma (CRC)-like immunoprofile (CK7−/CK20+/CDX2+/AMACR+/SATB2+); none of them was a CrD-SBA. No association between SATB2 expression and MMR status was observed. Although SATB2-positive SBA patients showed a more favorable outcome in comparison with SATB2-negative ones, the difference did not reach statistical significance. When cancers were stratified according to CK7/CK20 expression patterns, we found that CK7−/CK20- SBAs were enriched with MMR-deficient cases (71%) and patients with CK7−/CK20− or CK7−/CK20+ SBAs had a significantly better survival rate compared to those with CK7+/CK20− or CK7+/CK20+ cancers (*p* = 0.002). To conclude, we identified a small (6%) subset of SBAs featuring a full CRC-like immunoprofile, representing a potential diagnostic pitfall in attempts to identify the site of origin of neoplasms of unknown primary site. In contrast with data on colorectal carcinoma, SATB2 expression is not associated with MMR status in SBAs. CK patterns influence patient survival, as CK7−/CK20− cancers show better prognosis, a behavior possibly due to the high rate of MMR-deficient SBAs within this subgroup.

## 1. Introduction

Primary non-ampullary small bowel adenocarcinoma (SBA) accounts for less than 3% of malignant neoplasms of the gastrointestinal (GI) tract, despite the fact that the small bowel covers more than 90% of the whole GI surface area [1]. Nowadays, in the US population, SBA represents the second most common cancer histotype in the small bowel, following neuroendocrine tumors, and accounts for 31% to 40% of all small intestine malignancies [2]. Predisposing conditions for the SBA development include both hereditary syndromes and immune-mediated disorders, such as Crohn’s disease and celiac disease [3,4,5]. The duodenum is the most frequently involved segment (55–82%), followed by the jejunum (25–29%) and ileum (10–13%), the latter representing the predominant site in Crohn’s disease-associated SBAs [1,2]. Clinically, SBA is often detected at an advanced stage due to unusual and late onset of symptoms, poor endoscopic access, poor detection capability of the conventional radiological imaging and lack of an efficient and cost-effective screening method.

Histologically, SBA is a neoplasm similar to or indistinguishable from lower GI tract adenocarcinomas, although a higher incidence of poorly differentiated tumors has been described in SBAs [1]. SBAs, however, show some important epidemiological, clinical and molecular differences when compared with colorectal carcinomas (CRCs): (i) the incidence of CRC is declining, while that of SBAs is increasing [2]; (ii) SBA patients have a worse prognosis in comparison with CRC patients, as the five-year survival rate is 35% for SBA versus 51.5% for CRC [6]; (iii) unique molecular differences between SBA and CRC exist with regard to the percentage of several genetic driver mutations, such as *TP53* (58.4% in SBA vs. 75.0% in CRC), *APC* (26.8% vs. 75.9%) and *CDKN2A* (14.5% vs. 2.6%), leading to potential therapeutic implications [7].

In addition, higher rates of mismatch repair deficiency (MMR-d)/microsatellite instability have been reported in SBA in comparison to CRC, especially in early-stage disease [8]. Finally, although SBA treatments are usually extrapolated from CRC trials, recent studies have shown that the response to oncologic therapies in SBA patients is different from CRC cases; therefore, clinical practice guidelines specific for SBA have been published by a French intergroup in 2018 [9] and by the National Comprehensive Cancer Network (NCCN) in 2019 [10].

Clinico-radiologic and endoscopic distinction between SBA and CRC is usually straightforward; however, CRC may infiltrate or metastasize to the small bowel, mimicking a primary SBA, or be predominantly located around the ileocecal valve, simulating a primary neoplasm of the terminal ileum, especially in Crohn’s disease patients. In these challenging situations, as well as in the setting of metastasis of unknown primary origin, immunohistochemistry may play a certain role in suggesting the site of origin. The typical immunophenotypic profile of lower GI tract (colorectal or appendiceal) carcinomas is cytokeratin (CK)7−/CK20+/caudal-type homeobox 2 (CDX2)+, whereas about 60% of SBAs have been reported to co-express CK7 and CK20 and around 50% of SBAs are negative for CDX2 [11,12,13]. Nevertheless, on one hand, CK7 is also expressed in about 10% of CRCs [14] and CK20 and CDX2 markers can be lost in some CRCs, especially in those harboring MMR-d [15], while on the other hand, a fraction of SBAs (34%) have been reported to show a colorectal cancer-like immunohistochemical profile (CK7−/CK20+/CDX2+) [16].

Special AT-rich sequence-binding protein 2 (SATB2) is a known member of the AT-rich matrix attachment region binding transcription factor family with a role in central nervous system and craniofacial development, as well as osteoblast differentiation [17,18,19]. Within non-neoplastic cells of epithelial lineages, SATB2 expression is essentially restricted to glandular cells lining the lower GI tract (appendix, colon and rectum), in addition to cells lining the epididymis [20]. It is also positive in 83–85% of CRCs and in 75% of appendiceal adenocarcinomas, while it is uncommonly expressed in adenocarcinomas of gastric and pancreatic origin [20,21]. Therefore, it has been recently proposed as a new highly tissue-specific and sensitive marker of lower intestinal tract origin in both adenocarcinomas and well-differentiated neuroendocrine tumors [21,22]. However, a fraction (up to 46%) of SBAs have been reported to exhibit SATB2 positivity [16,21,23], although studies on SATB2 expression in SBA are limited to relatively small series, which include very few SBAs associated with Crohn’s or celiac diseases. Lastly, another potentially useful immunohistochemical marker for differential diagnosis between CRC and SBA is represented by alpha-methylacyl-CoA racemase (AMACR), a well-known biomarker for prostatic adenocarcinoma, as it has been observed in the majority of CRCs (62%), although it is rarely expressed (4%) in SBAs [24].

The aim of our study was to investigate the immunohistochemical expression of SATB2 and other GI phenotypic markers (CK7, CK20, CDX2, AMACR) in a large and etiologically well-characterized series of SBAs and to correlate the observed immunophenotypic profiles with clinico-pathologic and prognostic features, as well as with MMR status.

## 2. Results

### 2.1. Clinico-Pathologic Features of SBA Cases

In this study, we investigated a series of 100 surgically resected SBAs, most of which entered previous studies from the Small Bowel Cancer Italian Consortium [25,26,27,28,29]. Demographic and clinico-pathologic data are summarized in Table 1.

Our cohort included 34 Crohn’s disease-associated SBAs, 28 celiac disease-associated SBAs and 38 sporadic SBAs (Spo-SBAs). Mean age of enrolled patients at the time of cancer diagnosis was 63 years (range 27–95 years). Individuals were followed up for a median of 34 months (range 1–251 months) after surgery. The majority of patients were males, with a male-to-female ratio of 1.56:1. SBAs were located in the duodenum, jejunum or ileum in 11, 45 and 44 cases, respectively. When classified according to the American Joint Committee on Cancer (AJCC) grading system, 63 SBAs were low-grade, while 37 were high-grade. Overall, 11 cases were diagnosed in stage I, 49 in stage II, 31 in stage III and 9 in stage IV. According to histologic subtype classification, 70 SBAs showed a cohesive histotype (63 glandular-type and 7 medullary-type), while the remaining 30 cases featured a non-cohesive histotype (11 diffuse-type and 19 mixed-type). Although the majority of SBAs (63%) were MMR-proficient, a substantial number (37%) had a MMR-d phenotype. Among MMR-d SBAs, all cases but two showed a combined loss of MLH1 and PMS2; the remaining two cases, both associated with Crohn’s disease, harbored a combined loss of MSH2 and MSH6.

### 2.2. SATB2 Expression in SBAs and its Association with Clinico-Pathological Features

SATB2 immunostaining showed tumor cell immunoreactivity in 20 SBAs (20%), defined as weak, moderate and strong in ten, eight and two SBAs, respectively. Among the 20 SATB2-positive SBAs, the percentage of positive tumor cells ranged from 5% to 70%, with a mean of 27% positive tumor cells (mean H-score of 54). Of interest, two SATB2-negative SBAs showed SATB2 expression in their associated, adjacent dysplastic component. Among SATB2-positive SBAs, SATB2 expression in cancer-associated dysplasia was observed in only one case. Moreover, cancer-free ileal mucosa adjacent to two SATB2-negative Crohn’s disease-associated SBAs was seen to focally express SATB2. SATB2-positive cases included nine Spo-SBAs, seven celiac disease-associated SBAs and four Crohn’s disease-associated SBAs (Table 1). The prevalence of SATB2 positivity was lower in Crohn’s disease-associated SBAs (12%) in comparison with both celiac disease-associated (25%) or sporadic (24%) cases; however, the difference in the distribution of SATB2 expression amongst etiologic groups was not significant (*p* = 0.333). Expression of SATB2 was observed in 3 of 11 (27%) duodenal, 9 of 45 (20%) jejunal and 8 of 44 (18%) ileal cases, without statistically significant differences by tumor site (*p* = 0.797). No association was found between SATB2 positivity and patient age at cancer diagnosis, patient sex, AJCC tumor grade, stage, histotype or MMR status.

### 2.3. Association Between SATB2 and Other Phenotypic Marker Expression in SBAs

Positive CK20, CK7 and CDX2 immunostainings were observed in 62, 33 and 67 cases, respectively. No statistical association between the expression of CK20 or CK7 and SATB2 was found (*p* = 0.208 for CK20; *p* = 0.194 for CK7). On the other hand, the CDX2 intestinal marker was found to be expressed more frequently in SATB2-positive (17/20, 85%) than in SATB2-negative (50/80, 52%) SBAs, with a trend towards a statistical significance (*p* = 0.066). Stratifying the whole cohort according to the four possible CK7/CK20 expression patterns, we identified 12 CK7+/CK20+, 17 CK7−/CK20−, 21 CK7+/CK20- and 50 CK7−/CK20+ cases (Table 1). When comparing SATB2 expression among all four cytokeratin pattern-based groups, no significant association was observed (*p* = 0.243). Interestingly, we found a strong correlation between MMR status and CK7/CK20 immunophenotype (*p* = 0.007), as 71% of CK7−/CK20− SBAs had an MMR-d phenotype (Table 2).

**Figure 1:**
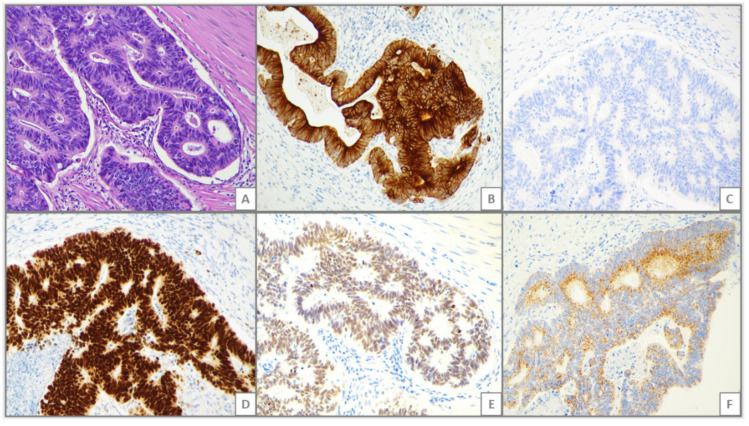
Representative histologic images of a small bowel adenocarcinoma (SBA) showing a full-blown colorectal cancer-like immunophenotype. (**A**) hematoxylin and eosin staining, showing a glandular-type SBA with well-formed glandular structures; (**B**) Cytokeratin (CK)20 immunohistochemistry, showing a strong and diffuse cytoplasmic reactivity for CK20; (**C**) CK7 immunohistochemistry, showing negativity for CK7 (**D**) Caudal type homeobox 2 (CDX2) immunohistochemistry, showing positivity for CDX2; (**E**) Staining for special AT-rich sequence-binding protein 2 (SATB2), showing SATB2 positivity; (**F**) Alpha-methylacyl-CoA racemase (AMACR), immunohistochemistry, showing AMACR tumor cell positivity. Original magnification: 200X.

A CK7−/CK20+/CDX2+ profile was detected in 46 cases and 14 of them (30%) co-expressed SATB2. Three of such 14 (21%) SBAs showed a SATB2 H-score >100. A CK7−/CK20+/CDX2+/SATB2+ pattern profile was present in three Crohn’s disease-associated SBAs, six Spo-SBAs and five celiac disease associated-SBAs. The CK7−/CK20+/CDX2+ immunoprofile was significantly more represented in SATB2-positive (14/20 cases, 70%) compared to SATB2-negative cases (32/80 cases, 40%; *p* = 0.023) (Table 1). When SATB2+ SBAs were also tested for AMACR expression, we identified a subset of six SBAs displaying an AMACR positive staining in addition to a CK7-/CK20+/CDX2+/SATB2+ phenotype (i.e., a full CRC-like immunoprofile). Clinico-pathologic and histologic features of these SBAs characterized by a CRC-like immunoprofile are reported in Table 3 and Figure 1. Among them, there were two celiac disease-associated SBAs and four Spo-SBAs; worthy of note, none of the three Crohn’s disease-associated SBAs having a CK7−/CK20+/CDX2+/SATB2+ pattern expressed AMACR. All cases were MMR-proficient and low-grade. The majority (five cases) showed a glandular histotype, whereas only one was a mixed type. All SBAs but one were alive after a median follow-up of 28 months; only one patient, with a low-grade, glandular-type, pT4, stage III SBA, died 3 months after surgery. In addition, two cases that were SATB2-positive but lacked a CK7−/CK20+/CDX2+ profile showed AMACR expression. One of these cases was a celiac disease associated-SBA, while the other was a Spo-SBA.

### 2.4. Survival Analysis

**Figure 2:**
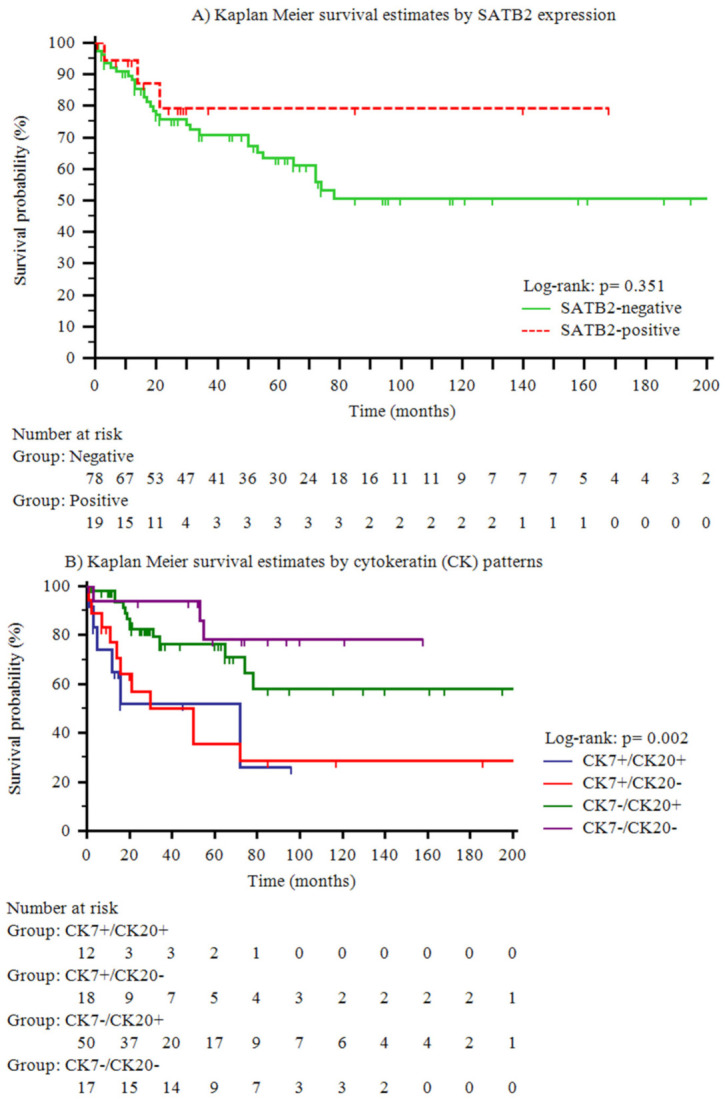
Kaplan–Meier cancer-specific survival estimates by special AT-rich sequence-binding protein 2 (SATB2) expression (**A**) and cytokeratin (CK) patterns (**B**).

Two patients died peri-operatively and one patient was lost to follow-up; therefore, they were excluded from survival analysis. Among the remaining 97 SBA patients, a trend towards a more favorable outcome of SATB2-positive SBA patients in comparison to those SATB2-negative was seen (Figure 2A). However, no statistically significant differences were found in cancer-specific survival between SATB2-negative versus SATB2-positive SBA patients, nor between SBAs with a CK7−/CK20+/CDX2+ or with CRC-like immunoprofile versus the remaining SBAs. On the contrary, CK patterns were found to be strongly associated with cancer-specific survival (*p* = 0.002), as patients with a CK7+/CK20+ or CK7+/CK20− pattern had a worse prognosis compared with those having a CK7−/CK20− or CK7−/CK20+ pattern (Figure 2B).

## 3. Discussion

In this study, we evaluated the expression of SATB2 and other immunophenotypic markers in a large SBA series of various etiologies. We found that 20% of SBAs expressed the lower GI marker SATB2, while 6% of SBAs exhibited a complete CRC-like immunoprofile (i.e., CK7−/CK20+/CDX2+/SATB2+/AMACR+).

SATB2 expression in SBAs has been previously described in smaller series [16,21,30]. The percentage of SATB2-positive SBAs in our series is significantly lower compared to that reported by Kim et al. (46%) [16] or Ma et al. (42%) [21]. Possible reasons for these discrepancies include more stringent selection criteria in our study (e.g., specific exclusion of cancers involving the ileo-cecal valve) and enrichment of cases associated with celiac or Crohn’s disease in our cohort. This is the first investigation of SATB2 expression in celiac disease-associated SBAs, reporting a 25% rate of positive cases. This finding may be of clinical interest because when a cancer of unknown primary is detected in a celiac patient, a SBA should not be excluded a priori even if it proves to be SATB2-positive, considering that celiac disease patients have a significantly higher risk for SBA development in comparison to the general population [31].

We observed that Crohn’s disease-associated SBAs were less frequently immunoreactive for SATB2 (12%) in comparison with sporadic (24%) or celiac disease-associated (25%) cases. This finding is interesting considering that Crohn’s disease-associated SBAs often express non-intestinal phenotypic markers, such as CK7 or the gastric foveolar marker MUC5AC [25,32]. In addition, the intestinal differentiation transcription factor CDX2 has been reported to be more frequently negative in Crohn’s disease-associated SBAs in comparison with celiac or Spo-SBAs [25]. In a recent study investigating CRCs associated with inflammatory bowel diseases (either Crohn’s disease or ulcerative colitis), the authors found that only 43% of colitis-associated carcinomas expressed SATB2, compared to 91% sporadic CRCs [33]. Taken together, these findings suggest that absence of SATB2 immunoreactivity cannot reliably exclude a colorectal or small bowel origin of a carcinoma of unknown primary in the patients affected by inflammatory bowel diseases.

In our study, the frequency of CK7, CK20 and CDX2 expression was similar to that reported by Kim et al. (33%, 62% and 67%, versus 52%, 70% and 72%, respectively) [16]. As expected, most SATB2-positive cases were also CDX2-positive (85%) and CK7-negative (80%); however, no statistically significant association between SATB2 and other individual phenotypic markers (CK7, CK20, CDX2) was found and only 25% of CDX2-positive SBAs were also SATB2-positive. In keeping with previous findings, CK7−/CK20+ was the most prevalent pattern also in our series, accounting for 50% of all cases, followed by CK7+/CK20− (21%), CK7−/CK20− (17%) and CK7+/CK20+ (12%) [16,34]. However, CK7+/CK20+ was reported to be the most common CK pattern in SBAs by other authors [11]. Interestingly, the CK pattern was associated with patient prognosis, showing patients with CK7−/CK20− tumors had a better survival rate, likely due to the very high rate of MMR-d (71%) within this subgroup, as well as among CK7− SBAs, which were associated with a better survival rate in comparison to CK7+ cancers [25]. In fact, MMR-d, which has already been reported to be negatively correlated with CK20 expression by SBA [34], represents an important favorable prognostic factor in SBAs, especially in stage II disease [29,35,36]. Therefore, SBAs in general and those showing a CK7−/CK20− profile in particular should be always tested for MMR protein expression. Furthermore, the positive prognostic role of CDX2 expression in SBAs has already been described [25,29,37].

Moreover, in keeping with the findings by Kim et al. [16], we found a significant fraction (46%) of SBAs showing a CK7−/CK20+/CDX2+ profile, which is typical of the vast majority (>90%) of CRCs. About 30% (14/46) of CK7−/CK20+/CDX2+ SBAs from our series also expressed SATB2, a percentage in between those reported by Bellizzi (21%) and Kim et al. (47%) [16,30]. However, in SATB2+ SBAs, we found a mean SATB2 H score of 54, similar to that reported in jejunal/ileal adenocarcinomas by Ma et al. (mean H score: 67), which was significantly lower in comparison with that of CRCs (mean H score: 128) [21]. In addition, SBAs with a SATB2 H-score >100 were rare (less than 10% in our series).

Six SBAs (6%) showed a CK7−/CK20+/CDX2+/SATB2+/AMACR+ immunoprofile (i.e., full CRC-like profile), suggesting that a small subset of SBAs fully resembling CRC immunophenotypically does exist. These cancers, all of which were low-grade and MMR-proficient and most of which showed a conventional glandular-type histology, seem to show a relatively less aggressive behavior. However, this finding needs to be further investigated in larger series. Importantly, none of these six cases occur in Crohn’s disease patients, while the four SATB2-positive Crohn’s disease-associated SBAs were all AMACR-negative, suggesting that an aberrant full-blown CRC-like phenotype is extremely rare in SBAs arising in Crohn’s patients.

Lack of SATB2 expression has been reported as an adverse prognostic factor in sporadic CRCs, even in those harboring MMR-d, while it was associated with lymph node metastases in colitis-associated CRC [33,38,39,40]. In our SBA series, we only found a non-significant trend towards a worse prognosis in SATB2-negative cases. In addition, we do not find any association between SATB2 expression and AJCC tumor stage or between SATB2 and tumor site, although previous studies reported a higher percentage of SATB2-positivity among jejunal/ileal cases compared to duodenal SBAs [16,21].

The molecular mechanism underlying aberrant SATB2 expression in a fraction of SBAs remains to be clarified. In contrast to findings in CRCs [21], we did not find any association between MMR status and SATB2 expression. Kim et al. reported three cases with SATB2 positivity in both the adenomatous and the adenocarcinomatous components, suggesting a potential role of SATB2 expression in small bowel adenoma initiation [16]. We also found SATB2-positive dysplastic components in three cases; however, two of them were SATB2-negative in their invasive component, indicating a marginal role of SATB2 expression in small bowel cancer development. Small bowel non-neoplastic mucosa adjacent to SBAs proved to be negative in most cases. However, two cases, both associated with Crohn’s disease, showed focal areas of SATB2-positive ileal epithelium, likely representing foci of metaplastic “colonic” phenotype of the ileal mucosa, which may occur in Crohn’s disease patients [41]. However, both SBAs originating in this background were SATB2-negative; thus, the contribution of this “colon-type” metaplastic phenomenon to small bowel carcinogenesis seems to be very limited.

## 4. Materials and Methods

### 4.1. Patients

This retrospective study included 100 patients with primary, non-ampullary, non-hereditary SBAs, confirmed by endoscopic and/or imaging techniques. Patients underwent surgical resection and had complete survival data from 24 tertiary referral Italian inflammatory bowel disease centers participating in the Small Bowel Cancer Italian Consortium. Diagnosis of celiac disease was based on serum IgA anti-endomysial and anti-tissue transglutaminase antibody positivity associated with typical duodenal histopathologic lesions [42]. Diagnosis of Crohn’s disease was ascertained according to international criteria [43] and the site and extent of the disease were confirmed by endoscopy, histology and imaging. SBAs infiltrating the colon or extending to the ileocecal valve as well as ampullary/periampullary cancers were rigorously excluded from analysis. This study was approved by the Ethics Committee of the Fondazione IRCCS San Matteo Hospital in Pavia (San Matteo Hospital Foundation Ethic Committee, protocol number 20140004197, 30 September 2014).

### 4.2. Histology and Immunohistochemistry

Tissue samples were fixed in 4% formaldehyde and embedded in paraffin wax. All SBA cases were investigated for histologic subtype, as previously described [25] and for all the parameters required by the eighth edition of AJCC TNM staging and grading system [44]. For immunohistochemistry, 4 μm-thick sections were stained on a Dako Omnis platform with the following antibodies: SATB2 (monoclonal, clone EPNCIR130A, Abcam, Cambridge, UK), CK7 (monoclonal, clone OV-TL 12/30, Dako Denmark A/S, Glostrup, Denmark), CK20 (monoclonal, clone Js20.8, Dako Denmark A/S, Glostrup, Denmark), CDX2 (monoclonal, clone DAK-CDX2, Dako Denmark A/S, Glostrup, Denmark), MLH1 (monoclonal, clone ES05, prediluted, Dako Denmark A/S, Glostrup, Denmark), MSH2 (monoclonal, clone FE11, prediluted, Dako Denmark A/S, Glostrup, Denmark), MSH6 (monoclonal, clone EP49, prediluted, Dako Denmark A/S, Glostrup, Denmark) and PMS2 (monoclonal, clone EP51, prediluted, Dako Denmark A/S, Glostrup, Denmark). In order to identify SBAs with a CRC-like immunoprofile, cases showing a positive nuclear staining for SATB2 were also tested for AMACR (monoclonal, clone 13H4, Dako Denmark A/S, Glostrup, Denmark). Cases featuring a CK7−/CK20+/CDX2+/SATB2+/AMACR+ immunohistochemical pattern were defined as having a CRC-like immunoprofile.

For the assessment of tumor cell immunophenotype, staining for CK7 and CK20 was considered positive when at least 10% of the neoplastic cells showed a cytoplasmic positivity, while CDX2-positive cases were defined as having nuclear staining in at least 20% of tumor cells, as previously reported [11,25]. SATB2 was scored considering both the percentage of neoplastic cells showing a nuclear positivity and the intensity of the staining. Cancers showing at least 5% of nuclear cells reactive for SATB2 were considered SATB2-positive [16]. The intensity was defined as 0 (absent), 1 (weak), 2 (moderate) and 3 (strong). In addition, H-score for SATB2, i.e., the intensity score multiplied for the percentage of cells with positive staining, was calculated to provide a SATB2 synthetic evaluation, taking into account both the intensity of expression and the density of positive cells [30]. Small bowel non-neoplastic mucosa adjacent to SBAs was also investigated for SATB2 expression. Cases were considered as positive for AMACR in the presence of a cytoplasmic staining in at least 5% of neoplastic cells [24].

Immunostaining of MMR proteins in tumor cells was considered as MMR-proficient if nuclear expression of all cancer cells was retained or MMR-d if nuclear expression was lacking, in the presence of an adequate internal positive control [25].

### 4.3. Statistical Analysis

The data were described with the mean and standard deviation if continuous and with counts and percentages if categorical; they were compared between groups with the Student’s *t*-test or the Fisher/Chi-square test, respectively. Median follow-up (25–75th percentile) was computed with the reverse Kaplan–Meier method. Follow-up was computed from diagnosis of cancer to death or last available follow-up for censored patients. Cumulative survival curves were plotted according to the Kaplan–Meier method and compared with the log-rank test. Stata 16.1 (StataCorp, College Station, TX, USA) was used for all analyses. A two-sided *p*-value of <0.05 was considered statistically significant.

## 5. Conclusions

Aberrant SATB2 expression and even a complete CRC-like immunoprofile may be observed in a fraction of SBAs, either sporadic or associated with immune-mediated intestinal conditions, in particular with celiac disease. While Crohn’s disease-associated SBAs may rarely express SATB2, a full-blown CRC-like immunoprofile is virtually absent in such a specific subset. Therefore, a small bowel origin cannot be definitely excluded in differential diagnosis of a cancer of unknown primary showing a CRC-like immunoprofile, especially when colonoscopy fails to reveal a CRC or the patient is affected by celiac disease. Likewise, SATB2 should not be considered a reliable marker to distinguish a CRC metastasis to the small bowel from a primary SBA infiltrating the large bowel, neither when coupled with AMACR expression. Although the association between SATB2 and outcome seems weak, CK7/CK20 expression efficiently stratifies SBA patient prognosis and may also be helpful to identify MMR-d cancers generally associated with a favorable outcome.

## Figures and Tables

**Table 1:** Demographic and clinico-pathologic features of small bowel adenocarcinoma patients by SATB2 expression.

Variable	Total	SATB2+	SATB2−	*p*-Value
**Number of cases**	100	20	80	
**Age at SBA diagnosis, years (mean + SD)**		63.6 ± 11.9	61.1 ± 15.3	0.497
**Male sex, *N* (%)**	61	14 (23)	47 (77)	0.446
**Site, *N* (%)**				0.797
Duodenum	11	3 (27)	8 (73)	
Jejunum	45	9 (20)	36 (80)	
Ileum	44	8 (18)	36 (82)	
**Etiology, *N* (%)**				0.333
Celiac disease	28	7 (25)	21 (75)	
Crohn’s disease	34	4 (12)	30 (88)	
Sporadic	38	9 (24)	29 (76)	
**Histologic subtype, *N* (%)**				0.277
Glandular	63	15 (24)	48 (76)	
Medullary	7	2 (29)	5 (71)	
Diffuse	11	0 (0)	11 (100)	
Mixed	19	3 (16)	16 (84)	
**Grade, *N* (%)**				0.496
Low	63	14 (22)	49 (78)	
High	37	6 (16)	31 (84)	
**AJCC stage, *N* (%)**				0.876
I	11	3 (27)	8 (73)	
II	49	10 (20)	39 (80)	
III	31	5 (16)	26 (84)	
IV	9	2 (22)	7 (78)	
**CK20+, *N* (%)**	62	15 (24)	47 (76)	0.208
**CK7+, *N* (%)**	33	4 (12)	29 (88)	0.194
**CK patterns, *N* (%)**				0.243
CK7+/CK20+	12	1 (8)	11 (92)	
CK7−/CK20+	50	14 (28)	36 (72)	
CK7+/CK20-	21	3 (14)	18 (86)	
CK7−/CK20-	17	2 (12)	15 (88)	
**CDX2+, *N* (%)**	67	17 (25)	50 (75)	0.066
**Profile CK7−/CK20+/CDX2+, *N* (%)**	46	14 (30)	32 (70)	0.023
**MMR-d, *N* (%)**	37	8 (22)	29 (78)	0.798

Legend: AJCC: American Joint Committee on Cancer; CK: cytokeratin; MMR-d: mismatch repair deficiency; MMR-p: mismatch repair proficiency; SBA: small bowel adenocarcinoma; SD: standard deviation.

**Table 2:** Relationship between expression of phenotypic markers and mismatch repair deficiency status in the 100 small bowel adenocarcinomas investigated.

Variable	No. of Cases (%)	MMR-p, *N* (%)	MMR-d, *N* (%)	*p*-Value
CK7 expression				0.028
CK7+	33 (33)	26 (79)	7 (21)	
CK7−	67 (67)	37 (55)	30 (45)	
CK20 expression				0.286
CK20+	62 (62)	42 (68)	20 (32)	
CK20−	38 (38)	21 (55)	17 (45)	
CK patterns				0.007
CK7+/CK20+	12 (12)	10 (84)	2 (16)	
CK7+/CK20−	21 (21)	16 (76)	5 (24)	
CK7−/CK20+	50 (50)	32 (64)	18 (36)	
CK7−/CK20−	17 (17)	5 (29)	12 (71)	
CDX2 expression				0.383
CDX2+	67 (67)	40 (60)	27 (40)	
CDX2−	33 (33)	23 (70)	10 (30)	

Legend: CK: cytokeratin; MMR-d: mismatch repair deficiency; MMR-p: mismatch repair proficiency.

**Table 3:** Clinico-pathologic features of the six small bowel adenocarcinoma (SBA) cases showing a colorectal carcinoma-like immunoprofile (CK7−/CK20+/CDX2+/SATB2+/AMACR+).

Case	Etiology	Age at SBA Diagnosis	Sex	Site	Histological Subtype	Grade	pT	AJCC Stage	CK20 (%)	CDX2 (%)	SATB2 (H-Score)	AMACR (%)	MMR-d	Status	Follow-up (mo)
#1	Sporadic	62	M	Ileum	Glandular	Low	4	3	40	90	100	70	No	DOD	3
#2	Sporadic	65	M	Jejunum	Glandular	Low	3	2	60	90	25	40	No	Alive	37
#3	Sporadic	53	F	Ileum	Glandular	Low	3	2	25	90	210	15	No	Alive	28
#4	Sporadic	82	M	Jejunum	Glandular	Low	3	3	80	80	20	20	No	Alive	11
#5	Celiac	65	F	Duodenum	Glandular	Low	2	1	60	100	10	70	No	Alive	30
#6	Celiac	83	M	Jejunum	Mixed	Low	3	3	70	100	80	80	No	Alive	12

Legend: AJCC: American Joint Committee on Cancer; AMACR: alpha-methylacyl-CoA racemase; CDX2: caudal type homeobox 2; CK20: cytokeratin 20; DOD: dead of disease; F: female; M: male; MMR-d: mismatch repair deficiency; mo: months; SBA: small bowel adenocarcinoma.
